# Geographical Origin Traceability of *Procambarus clarkii* Based on Mineral Elements and Stable Isotopes

**DOI:** 10.3390/foods11193060

**Published:** 2022-10-01

**Authors:** Yun Xia, Lijuan Jia, Kai Zhang, Jun Xie, Ermeng Yu, Jingjing Tian, Wangbao Gong, Zhifei Li, Hongyan Li, Guangjun Wang, Yarong Liu

**Affiliations:** Key Laboratory of Tropical & Subtropical Fishery Resource Application & Cultivation, Ministry of Agriculture, Pearl River Fisheries Research Institute, Chinese Academy of Fishery Sciences, Guangzhou 510380, China

**Keywords:** *Procambarus clarkii*, geographical origin traceability, mineral elements, stable isotope

## Abstract

We explore the prospect of applying mineral element and stable isotope data in origin tracing *Procambarus clarkii* to establish an origin tracing system. Microwave digestion–atomic absorption spectrometry and stable isotope ratio mass spectrometry determined the contents of 14 mineral elements (Na, Mg, Al, K, Ca, Mn, Zn, Cu, Fe, Sr, Ba, As, Se and Cd) and the abundances of C and N stable isotopes in the muscle tissue of *P. clarkii* from Guangdong, Hunan and Hubei regions. The one-way ANOVA and Duncan multiple comparison results revealed Na, Sr, Ba, Cu, Mn, Fe, Al, Se, δ^13^C and δ^15^N varied significantly between the three regions (*p* < 0.05). A systematic clustering analysis revealed the stable isotopes combined with the mineral elements easily distinguished samples into the three different regions. Multivariate statistical analysis allowed us to establish a discriminant model for distinguishing *P. clarkii* from the three geographical regions. When stable isotopes were combined with mineral elements, the accuracy of the linear discriminant analysis of the samples from Guangdong, Hunan and Hubei were 95%, 95% and 100%, respectively. The initial overall discriminant accuracy was 96.7%, and the cross-validation discriminant accuracy was 93.3%. Principal component analysis identified three main components which were based on eleven major factors, including Cu, Ba, Cd, Mn, δ^13^C, δ^15^N, Al and Mg, resulting in a cumulative variance contribution rate of 78.77%. We established a three-dimensional coordinate system using the three principal components to create scatter diagrams with the samples from the three regions in the coordinate system. The results revealed the samples clearly differentiated into the three regions. Therefore, mineral elements combined with stable isotopes can distinguish the regional origin of *P. clarkii*.

## 1. Introduction

*Procambarus clarkii*, also known as crayfish and red crayfish, is widely appreciated by consumers because it is delicious and highly nutritious. *P. clarkii* is omnivorous, fast growing and has a high adaptability. These combined traits are competitive advantages in local ecological environments. *P. clarkii* is native to North America and was first developed and utilized as an aquatic resource in China in 1983. Since 2007, *P. clarkii* aquaculture in China has expanded swiftly, reaching a production of approximately 2.39 million tons in 2020, and is now ranked sixth in the China’s freshwater aquaculture species [1]. *P. clarkii* is favoured by the younger generation of consumers, and there has been a trend of explosive growth in the catering consumption market. The breeding of *P. clarkii* mainly occurs in Hubei, Hunan, Anhui, Jiangsu and Jiangxi provinces [1]. In Jiangsu province, Xuyi lobster (local name for *P. clarkia*) is famous for its pure ecology, tender meat, good quality, good taste and impressive market reputation [2]. Qianjiang and Jingzhou in Hubei province are famous crayfish farming areas with the largest crayfish production [2]. Consumer feedback has been consistent over many years, the crayfish cultured in Jiangsu are relatively good in taste and size, especially crayfish from Xuyi. In order to improve the social and economic benefits of agricultural products, the Chinese government is implementing the “protection of geographical indication products”. In recent years, great importance has been attached to the brand construction of *P. clarkii*, especially to the construction of public regional brands with wide coverage and strong effective drivers. In 2020, the construction of the public brand of *P. clarkii* was ushered in the outbreak period and added nine geographical indication certification trademarks (Xiangyang Crayfish, Huarong Crayfish, Majiadang Crayfish, Kaijiang Crayfish, Hanshan Crayfish, Gongqingcheng Crayfish, Junshan Crayfish, Yanjiang Zhonghe Crayfish and Xinghua Crayfish). Until recently, 21 brands of *P. clarkii* origin were under the protection of the national geographical indication certification trademark. The geographical indication of a product and the agricultural product geographical indication certification plays a protective role for well-known brands of *P. clarkii*.

However, the internet and/or the aquatic products market allows various aquatic products to be provided with the logo of “origin”. Unfortunately, fake brands can be sold by unscrupulous merchants in order to obtain higher profits. This practice not only damages the interests of consumers and local farmers, but also poses a serious threat to regional brands. The aquaculture market needs regulation to protect the legitimate rights and interests of consumers and farmers. It is particularly important to establish a perfect anti-counterfeiting discrimination system for *P. clarkii* to trace the source of counterfeit and shoddy products. However, information is often lost in practical production and application. This can be due to lost labels and false labelling and other reasons which can result in fake and shoddy products. Therefore, easily identifiable and independent methods are needed to effectively identify the origins of crayfish products, to effectively supervise the market.

Traceability provides aquatic product safety management, and origin traceability is an important part of it. Origin tracing has many advantages, including that it is conducive to securing the rights and interests of consumers, protecting regional products, assuring fair competition, and effectively recalling products in the event of food safety incidents such as environmental pollution or disease epidemics [3]. The traditional method of aquatic product origin tracing is to physically add an anti-counterfeit label [4]. This method works to a certain extent to distinguish the different product sources, but this kind of anti-counterfeiting mark is independent of the product and easily lost or damaged in processing or transportation, which then uncouples the information chain. In addition, the use of false labels undermines anti-counterfeit labels. It is imperative to develop a new method which combines the origins with the environmental characteristics and dietary characteristics of aquatic products. Tracing the origin of aquatic products may be possible using mineral elements and stable isotope abundances [5]. At present, mineral elements and stable isotope technologies have been used to trace the origins of agricultural products, such as tea [6,7], meat and mutton [8,9,10], as well as aquatic products, such as *Eriocheir sinensis* [5,6,7,8,9,10,11] and the swimming crab [12]. Nevertheless, no research has been published on tracing *P. clarkii* origins.

The contents of 14 mineral elements and the abundance of C and N stable isotopes in the muscles of *P. clarkii* from Qingyuan (Guangdong province), Hengyang (Hunan Province) and Qianjiang (Hubei Province) were compared. We aim to establish a regional discrimination model for *P. clarkii*, by screening the characteristic indexes which distinguish different regions to enable origin traceability for *P. clarkii*.

## 2. Materials and Methods

### 2.1. Sample Collection

*P. clarkii* (*n* = 100) were collected in July 2021 from the Qingyuan rice–crayfish integrated culture commercial farm in Guangdong (GD), the Hengyang rice–crayfish integrated culture commercial farm in Hunan (HN) and the Qianjiang rice–crayfish integrated culture commercial farm in Hubei (HB). Juvenile crayfish (avg. length 4.71 ± 0.34 cm, avg. weight 8.93 ± 0.56 g) from the three farms were obtained from the HAID Group Co., Ltd. (Guangdong, China), in April 2021. The farming management in the three farms was similar, and all the farms used the same brand and same batch of commercial feeds (HAID Group Co., Ltd., crude protein 30.0%, crude fat 3.0%). The water quality parameters of the three regions are shown in Table 1. In addition, the δ^13^C and δ^15^N values and contribution rates of food sources for *P. clarkii* in these three regions were analyzed, which indicated that the food sources of crayfish mainly included POM, sediment debris, rice straw, benthos, commercial feed, etc., (unpublished data). For each location, 100 commercially sized crayfish were selected based on their similar size, with an equal number of male and female specimens to reduce the effects of intrinsic factors (e.g., species, size, age and/or sexual maturity) [13]. The parameters investigated included body length and body weight (Table 2). For each location, we mixed five crayfish of the same gender as one sample in order to create 10 male samples and 10 female samples for each location, and a total of 60 samples were collected for each of the 3 locations. The abdominal muscles of *P. clarkii* were homogenously mixed and dried in a vacuum freeze-dryer (FD-1, China) for 72 h, before being ground through a 100-mesh sieve and stored in a dryer until required for the analyses.

### 2.2. ICP-MS Analysis

Element compositions of the samples were detected using inductively coupled plasma mass spectrometry (ICP-MS) (Agilent 7500ce, Agilent Technologies Inc., Santa Clara, CA, USA) in the China National Analytical Center, Guangzhou. A total of 14 elements (including K, Ca, Na, Mg, Mn, Zn, Cu, Al, Fe, Sr, Ba, As, Se and Cd) were determined. The detailed detection and validation methods are described in Luo et al. [14]. The dry muscle samples (0.5 ± 0.001 g) were treated with 5 mL nitric acid (GR, Merck Inc., Darmstadt, Germany). Each sample mixture was gently digested using a microwave digestion instrument (PreeKem COOLPEX, Shanghai, China). The cooled digests were diluted to 25 mL with ultra-pure water prior to analysis using an ICP-MS. The linear correlation coefficients of the analysed elements were all >0.999. The recovery of all the elements in this study ranged from 96.5 to 112.9% (*n* = 20). The detection limits of the methods were 1.0 mg/kg for K, 1.0 mg/kg for Ca, 1.0 mg/kg for Na, 1.0 mg/kg for Mg, 0.1 mg/kg for Mn, 0.50 mg/kg for Zn, 1.0 mg/kg for Fe, 0.05 mg/kg for Cu, 0.5 mg/kg for Al, 0.2 mg/kg for Sr, 0.02 mg/kg for Ba, 0.002 mg/kg for As, 0.01 mg/kg for Se and 0.002 mg/kg for Cd.

### 2.3. Isotope Analyses

The stable isotope ratios of carbon and nitrogen in the muscle samples were analysed in the South China Sea Institute of Oceanology of CAS following the methods described in Luo et al. [14]. The details are as follows: a small quantity (2 mg) of the dry sample was placed in a tin cup and the δ^13^C and δ^14^N values were measured using a coupled element analyser (EA IsoLink; Thermo, Waltham, MA, USA) and a stable isotope ratio mass spectrometer (DELTA V Advantage; Thermo, Waltham, MA, USA), respectively. The carbon/nitrogen stable isotope natural abundance is expressed, as Anderson et al. [15]:

δX = [(R_sample_/R_standard_) − 1] × 1000% (1)

where X represents ^13^C or ^15^N, and R_sample_ represents the ^13^C/^12^C ratio for C or the ^15^N/^14^N ratio for N. The accuracies of the δ^13^C and δ^15^N isotopic analyses were assessed using a commercially available isotope standard (IAEA-600 Caffeine) with certified δ^13^C values and an informative δ^15^N value of −27.771 Vienna Pee Dee Belemnite standard (VPDB) and +1.0‰ atmospheric N_2_ (air N_2_), respectively. The standard deviations (precision) of the analysis using IAEA-600 (caffeine) were, 0.1‰ for δ^13^C and 0.2‰ for δ^15^N, with ten replicates. For quality control, calibration with standards was performed after every 10 samples. For data correction, the reference gas was recalibrated, and the samples were reanalysed if the IAEA-600 values were outside the range of the reference values.

### 2.4. Statistical Analysis

The between regional variation of the stable isotope and mineral element contents were analysed by one-way ANOVA and Duncan’s test using SPSS 19.0 (SPSS, Inc., Chicago, IL, USA). In addition, a cluster analysis, linear discriminant analysis (LDA) and principal component analysis (PCA) were undertaken using the stable isotope ratio values and the 14 mineral element contents to determine whether these factors can be used to distinguish crayfish from different regions. In all analyses a probability value less than 0.05 was considered statistically significant (*p* < 0.05).

## 3. Results

### 3.1. Analysis of Origin Variation in Mineral Elements and Stable Isotopes

The contents of 14 trace elements (K, Ca, Na, Mg, Mn, Zn, Cu, Al, Fe, Sr, Ba, As, Se, Cd) in the 60 samples collected from three different regions were measured. The details are summarized in Table 3. The four macronutrients (Ca, K, Mg and Na) accounted for more than 99% of the samples. The remaining elements amounted to less than 1%. Among the four main elements, the K content of HN was significantly higher than GD (*p* < 0.05). However, there was no significant variation in K content between HN and HB or between GD and HB (*p* > 0.05). The Na content in HN was significantly higher than in HB or GD, and significantly higher in HB than in GD (*p* < 0.05). The Mg contents in HN and HB were significantly higher than in GD (*p* < 0.05), but there was no significant variation between HN and HB (*p* > 0.05). The Ca contents in GD and HB were significantly higher than in HN (*p* < 0.05), but there was no significant variation between GD and HB (*p* < 0.05). Among the remaining elements, the contents of Mn, Cu, Sr, Ba, Se and Cd in HN were significantly higher than in GD or HB (*p* < 0.05). The contents of Ba, Mn, Se and Cu in GD were significantly higher than in HB (*p* < 0.05). The Sr contents in GD were significantly lower than in HB (*p* < 0.05). There was no significant variation in the Cd contents between GD and HB (*p* > 0.05). The contents of Fe, As, Al and Zn in GD were significantly higher than in HN (*p* < 0.05). The contents of Fe and Al in HN were significantly higher than in HB (*p* < 0.05). The Zn content in HN was significantly (*p* < 0.05) lower than in HB (*p* < 0.05). There was no significant (*p* > 0.05) variation in Fe, Al or Zn between GD and HB (*p* > 0.05). The content of As in GD was significantly higher than in HB (*p* < 0.05), but there was no significant variation between HN and HB (*p* > 0.05).

**Figure 1:**
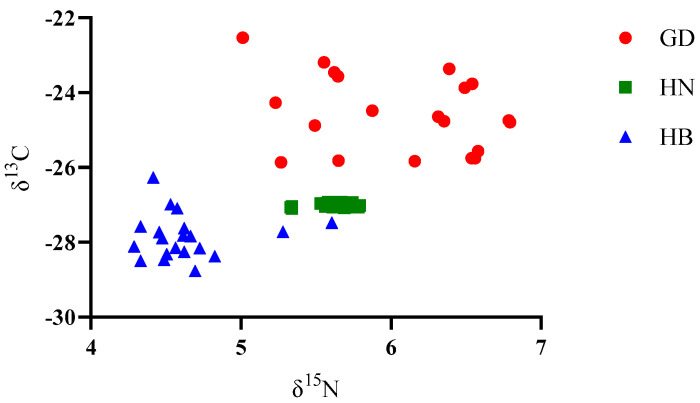
Scatter plot based on δ^13^C and δ^15^N of 60 samples. GD = Guangdong; HN = Hunan; HB = Hubei. δ^13^C and δ^15^N represent the stable isotope ratios.

**Figure 2:**
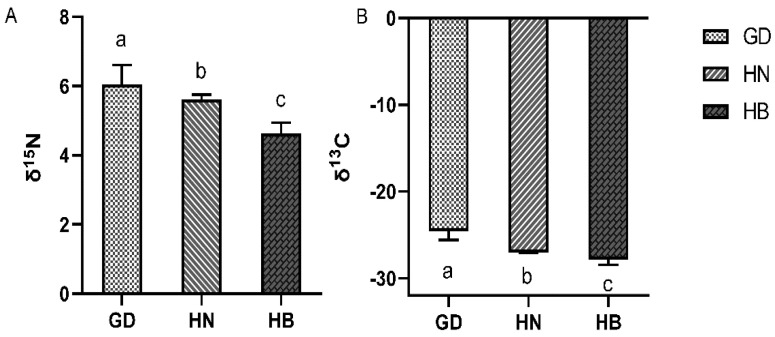
Stable isotope results from *P. clarkii* muscle samples. GD = Guangdong; HN = Hunan; HB = Hubei. δ^15^N results (**A**); δ^13^C results (**B**). δ^13^C and δ^15^N represent the stable isotope ratios. The different superscript lowercase letters (a, b, c) indicate a significant difference at *p* < 0.05 based on a one-way ANOVA and Duncan’s test.

As shown in Figure 1, the scatter plot based on δ^13^C and δ^15^N of the 60 samples showed that each group of samples can be well differentiated, and all δ^13^C values ranged from −28.77‰ to −23.19‰, whereas the δ^15^N values ranged from 4.29‰ to 6.79‰. Among these, *P. clarkii* from GD exhibited significantly higher δ^13^C and δ^15^N than HN or HB (*p* < 0.05) (see Figure 2). Additionally, the δ^13^C and δ^15^N values of HN were significantly higher than HB (*p* < 0.05).

### 3.2. Systematic Clustering Analysis

**Figure 3:**
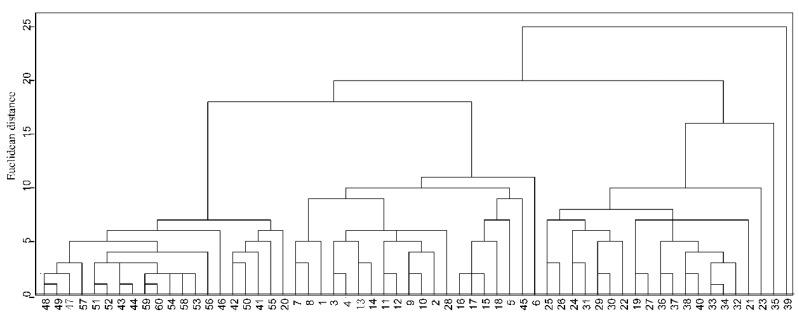
Systematic clustering results for *P. clarkii* muscle samples from three different regions. Samples 1–20 are from Guangdong; samples 21–40 are from Hunan; samples 41–60 are from Hubei.

Combining the element compositions with the stable isotope ratio values in the cluster analysis of *P. clarkii* resulted in the samples being divided into three categories when the Euclidean distance was 10 (Figure 3). There were 20 samples within the first category, including 19 HB samples and 1 GD sample. There were 19 samples in the second category, including 17 GD samples, an HN sample and an HB sample. The third category contained 18 samples, including 12 HN samples and a GD sample. Additionally, one GD sample and two HN samples were wrongly classified into another subclass. The results of the systematic clustering analysis revealed stable isotopes combined with mineral element data provides excellent clustering of the samples into their regional origin. Only a few samples were incorrectly classified.

### 3.3. Linear Discriminant Analysis

**Figure 4:**
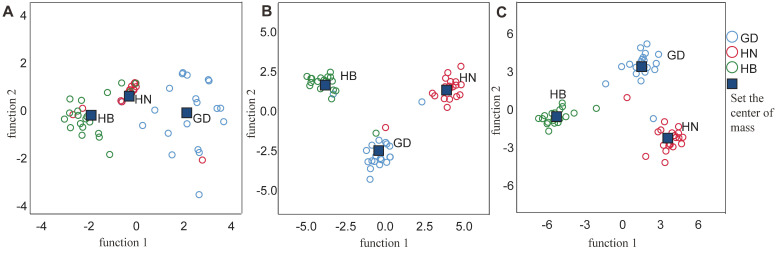
Scatter plots of the linear discriminant analysis of *P. clarkii* sampled from different regions. The scatter plot of δ^13^C and δ^15^N (**A**). The scatter plot of 14 elements (**B**). The scatter plot of 14 elements combined with the stable isotope data (**C**). δ^13^C and δ^15^N represent the stable isotope ratio. GD = Guangdong; HN = Hunan; HB = Hubei.

The LDA analysis of the δ^13^C with δ^15^N and 14 element values revealed comprehensive original discriminant accuracies of 83.3% and 93.3% (Table 4), respectively. The scatter plot of δ^13^C and δ^15^N showed that all three of the tested regions could be identified, with minor overlap with discriminant accuracy rates of 80% (i.e., the Hubei region) to 85% (Figure 4A, Table 4A). The samples from GD were more variable than those from the other regions. Both the HB and HN samples clustered with their location of origin. The scatter plot of the 14 element results shows the distribution of the samples from the three regions could be clearly identified with limited overlap, with discriminant accuracy rates of 90% to 95% (Figure 4B, Table 4B).

Notably, when the element compositions were combined with the values of the stable isotope ratios, the original discrimination rate reached 96.7% (and a cross-validated rate of 93.3%) and all three regions were clearly distinguished using two discriminant functions for all three groups (Figure 4C, Table 4C). This result reveals the combined chemical data sets enhanced the discrimination accuracy.

### 3.4. Self-Organizing Neural Network Analysis

**Figure 5:**
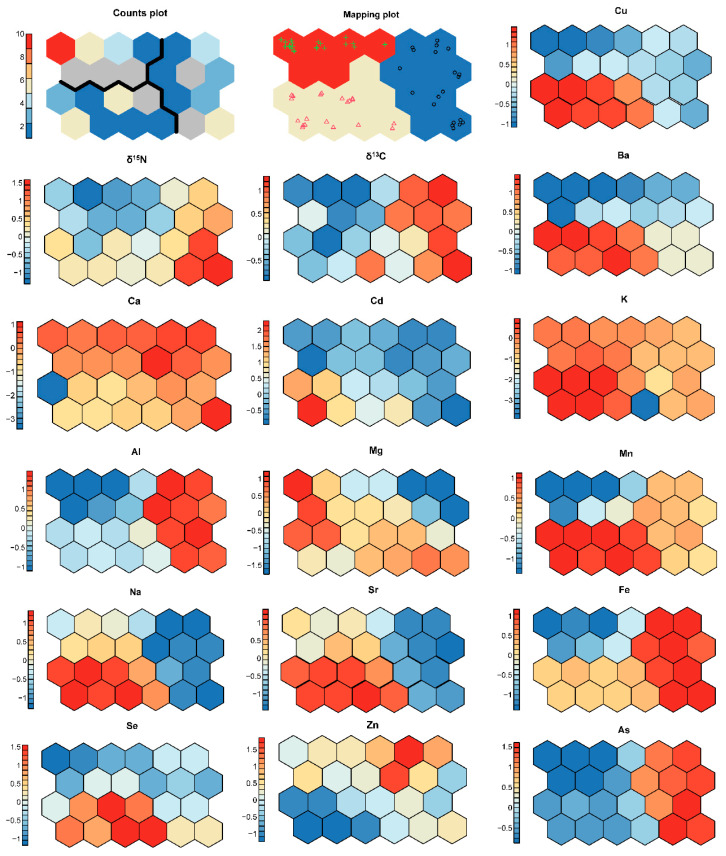
Visual analysis of the component planes. The closer the colour is to red, the higher the relative content; the closer the colour is to blue, the lower the relative content.

Self−organizing mapping (SOM) visually displayed the distribution of the 16 components from the three different regions (Figure 5). The 16 components separated the samples into the three different regions. The mineral elements and stable isotopes could identify which region a sample originated from. Among these, Cu, Ba and Mn have similar characteristics, with higher levels in HN than HB and GD. However, Ca and Zn values had different distribution characteristics which were consistent with the remaining components.

### 3.5. Principal Component Analysis

**Figure 6:**
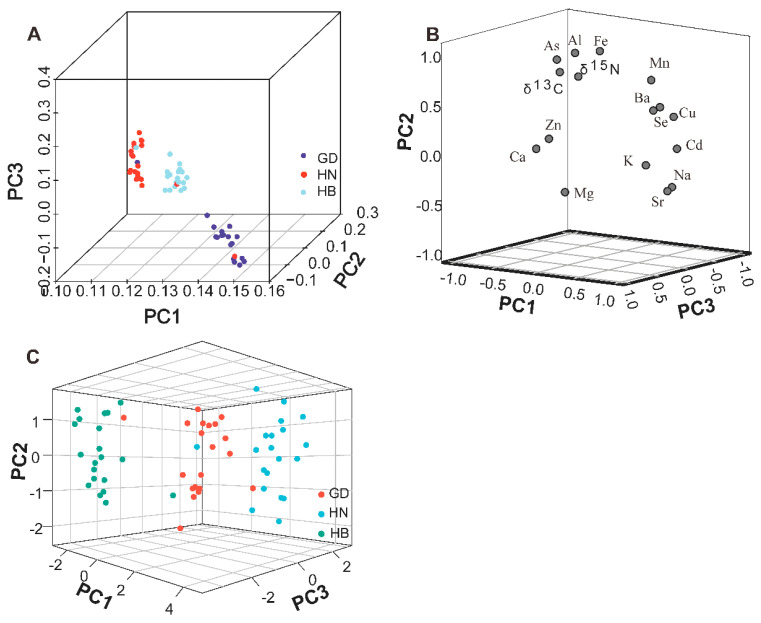
Principal component analysis of *P. clarkii* variables sampled from different regions. PCA score plot combines the element compositions with the stable isotope ratios in the variables (**A**). Distribution diagram of the main elements influencing the principal components (**B**). Three-dimensional scatter diagram of the samples based on the main contributing elements of the principal components (**C**).

Principal component analysis was performed on the 14 element concentrations and the ratios of the stable isotopes to investigate the potential of discriminating between *P. clarkii* origins. The first three principal components explained 78.77% of the variance (component 1 explained 40%, component 2 explained 32.34% and component 3 explained 3.42%) of the total variability and were used to draw a three−dimensional scatter plot (Figure 5). As shown in Figure 6A, the PCA score plot illustrates a clear separation pattern with *P. clarkii* origin when the variables are based on the element compositions with the stable isotope ratios. As Figure 6B illustrates, elements Cu, Ba, Cd and Mn were the dominant variables on the first principal component. The second principal component was strongly associated with the values of the δ^13^C, δ^15^N and Al. The third principal component was mainly based on Mg. The eight main contributing elements in the principal components were used to make a three-dimensional scatter plot of the *P. clarkii* samples. The principal components clearly separated the samples into their region of origin (Figure 6C). Given these results, it is apparent that Cu, Ba, Cd, Mn, δ^13^C, δ^15^N, Al and Mg values can be used to determine *P. clarkii* origin.

## 4. Discussion

In the natural state, the stable isotope compositions within organisms vary with the natural fractionation effects of climate, environment and metabolic type [16,17]. These natural attributes can discriminate between regions, allowing them to be used for origin tracing. Stable isotopes, such as carbon and nitrogen required for the growth of aquatic animals, all come from the assimilation process of ingested food. Guo et al. traced the origins of wild and artificially cultured mangrove blue crabs by using the stable isotopes of carbon and nitrogen and found significant differences in carbon and nitrogen stable isotopes, which was related to the stable isotopic composition of food sources in the habitats [18]. Carrera et al. [19] found that the δ^13^C and δ^15^N in hake species from six different fishing areas were significantly different, allowing them to be used for origin tracing. Peng et al. [12] showed that carbon and nitrogen stable isotopes in tissue from *Portunus trituberculatus* collected from three different habitats varied significantly, allowing the discrimination of *P. trituberculatus* grown in different waters, and the muscle tissues performed better than the gill or liver tissues in the discrimination model. In this research, the stable carbon and nitrogen isotopes in muscle from crayfish can discriminate between regions, and this was related to the different natural food sources of crayfish in different regions (unpublished data).

The scatter plot based on δ^13^C and δ^15^N showed that each group of samples can be well differentiated in this research. The 1δ^13^C values ranged from −28.77‰ to −22.53‰, and δ^15^N ranged from 4.29‰ to 6.79‰, which may reflect significant differences in the food sources of the different regions. Both δ^13^C and δ^15^N were the highest in *P. clarkii* samples from Guangdong and samples from Hubei were the lowest. This result shows the effects on stable isotopes of *P. clarkii* living in different environments. The main reason for the variation in δ^13^C and δ^15^N is the different diets [20]. Carbon isotopes are closely related to aquatic animal diets and represent the proportion of C3 and C4 plants within the feed. Nitrogen stable isotopes are related to the animal trophic levels and the use of nitrogen and nitrogen fertilizer in soil. The original discrimination accuracy was up to 83.3%, and the cross-validation accuracy was up to 83.3% in this research. There was an excellent discrimination in the carbon and nitrogen stable isotopes for tracing the origins of the *P. clarkii* samples, but the specific reasons for this difference need to be further clarified.

Organisms cannot synthesize mineral elements by themselves, and the mineral elements in their bodies come from their daily diet and living environment [21,22]. There is a correlation between diet and environment that provides a theoretical basis for tracing animal origins using mineral elements. The compositions and mineral element contents in soil, water, feed, waste and air of the rearing environment have their own characteristics [21]. Elements in the rearing environment continuously accumulate in an organism, resulting in large variation between organisms from different origins [23]. Artificial factors such as the feed type, feed additives, fertilizer and/or fertilization frequencies all affect or offset variations in the elements in different regions. Guo et al. [24] detected the contents of 25 metal elements in economic fish from three regions of the East China Sea and used partial least squares discriminant analysis to classify them, resulting in a discrimination accuracy rate of 97.92%. In this study, the scatter plot of 14 mineral elements showed that the geographical locations could be clearly distinguished, suggesting these elements can be used in the origin tracing of *P. clarkii*. The origin discriminant analysis of *P. clarkii* samples was carried out using 14 mineral elements, resulting in a 93.3% accuracy of both the original discriminant and the cross-validation, indicating excellent discrimination.

The stable isotope ratio and mineral element contents can be used to trace the origins of aquatic products [25,26]. Carbon and nitrogen stable isotopes and mineral elements have been widely used in sourcing the origin of some aquatic animals, including *E. sinensis* [14], Brazilian freshwater catfish [27] and salmon [28]. In this study, the results of the carbon and nitrogen discrimination accuracy was 83.3%, indicating that these factors can distinguish between the three regions, with some inaccuracies. Therefore, additional stable isotopes need to be included in the detection or the carbon and nitrogen stable isotopes need to be combined with other traceability methods to obtain higher discrimination accuracy. The combination of mineral elements and stable isotopes improved the stability of the discriminant model [5]. Curtis et al. [29] studied the otolith structure of sea salmon using stabilizing isotopes with mineral elements and showed that it can be used as a powerful tool to distinguish spawning fields in wild spotted sea salmon. Ortea et al. [30] achieved accurate origin discrimination of prawns by combining mineral elements with stable isotopes. Luo et al. [14] traced the origin of *E. sinensis* using a combination of mineral elements and stable isotopes. In this research, similar to the findings of Zhang et al. (2020) [5], by combining the stable isotope ratio with the mineral element content, the origin of *P. clarkii* from the three regions had a discrimination accuracy of 96.7% and a cross-validation accuracy of 93.3%, indicating the model had higher accuracy and stability. Taken together, these results show that either using 14 mineral elements or combining stable isotope and mineral elements can produce a better discrimination effect. Combining stable isotopes with mineral elements can improve the accuracy of a discrimination model.

Multivariate statistical analysis investigated the correlations between multiple objects and included a cluster analysis, linear discriminant analysis and principal component analysis [31], these tools have been widely used in food origin traceability [32,33]. The variables consisted of the two stable isotopes and 14 mineral elements and were used to conduct the ANOVA and Duncan multiple comparison of *P. clarkii* in integrated rice–crayfish breeding systems in the Guangdong, Hunan and Hubei regions. Each factor varied (to different degrees) between the regions. The results of cluster analysis, self-organizing neural network analysis and LDA were effective in identifying the provenance of *P. clarkii* with the three regions. Samples from three regions were divided into three groups by the clustering analysis and the self-organizing neural network analysis with only a few samples being incorrectly classified. The original discrimination rate of the LDA of the three regional samples was 96.7%, and the cross-validation rate was 93.3%. These results indicate that this model had an excellent discriminating effect with high accuracy and stability. Finally, three principal components were created with the 16 factors using a PCA. The three principal components successfully divided all the test samples into the three different regions. Cu, Ba, Cd, Mn, δ^13^C, δ^15^N, Al and Mg were the characteristic indexes within the principal components which distinguished the sample origins in the discrimination model.

## 5. Conclusions

We measured 14 mineral elements and the values of δ^13^C and δ^15^N in *P. clarkii* muscle. The data was analysed using multivariate statistical analysis to identify individual *P. clarkii* origins. The clustering analysis (using SOM, LDA, and PCA) revealed both the mineral elements and the mineral elements combined with stable isotopes could be used to trace *P. clarkii* origins. Specifically, Cu, Ba, Cd, Mn, δ^13^C, δ^15^N, Al and Mg are characteristic indexes for origin identification. Both methods (the mineral elements or mineral elements combined with stable isotopes) established by this study provide important base references and theoretical support for the protection and identification of *P. clarkii* geographical identification of products. The next stage of this research involves collecting samples from other regions to verify our research method in our pursuit of establishing a mature and stable identification technology for *P. clarkii* origin.

## Figures and Tables

**Table 1:** The water quality parameters in different regions (*n* = 6).

Water Quality Index	GD	HN	HB
T/(°C)	31.05 ± 0.15	27.84 ± 0.18	35.66 ± 0.40
DO/(mg/L)	4.84 ± 0.14	3.85 ± 0.19	3.26 ± 0.14
SPC/(μs/cm)	191.65 ± 4.74	174.5 ± 6.31	94.93 ± 1.80
TDS/(mg/L)	122.07 ± 2.25	110.36 ± 1.95	60.48 ± 1.68
pH	8.61 ± 0.09	7.02 ± 0.07	9.64 ± 0.06
CODcr/(mg/L)	24.70 ± 3.24	31.70 ± 2.63	73.20 ± 3.97
TSS/(mg/L)	21.00 ± 1.36	13.80 ± 1.19	45.70 ± 1.65
Chl.a/(μg/L)	0.027 ± 0.002	0.024 ± 0.002	0.037 ± 0.001
TP/(mg/L)	1.72 ± 0.05	0.71 ± 0.04	1.29 ± 0.04
PO_4_^3−^-P/(mg/L)	0.41 ± 0.02	0.21 ± 0.02	0.24 ± 0.02
NH_4_^+^-N/(mg/L)	0.13 ± 0.004	0.20 ± 0.007	0.26 ± 0.009
NO_3_^−^-N/(mg/L)	0.21 ± 0.02	0.40 ± 0.02	0.18 ± 0.02
NO_2_^−^-N/(mg/L)	0.022 ± 0.001	0.037 ± 0.002	0.017 ± 0.001
TN/(mg/L)	2.88 ± 0.11	2.85 ± 0.10	4.65 ± 0.12

GD = Guangdong; HN = Hunan; HB = Hubei; T = Water Temperature; DO = Dissolved Oxygen; SPC = Specific Conductance; TDS = Total Dissolved Solids; pH = pH value; CODcr = Dichromate index; TSS = Total Suspended Solids; Chl.a = Chlorophyll A; TP = Total Phosphorus; PO_4_^3−^-P = Orthophosphate; NH_4_^+^-N = Ammonia Nitrogen; NO_3_^−^-N = Nitrate; NO_2_^−^-N = Nitrite; TN = Total Nitrogen.

**Table 2:** Basic weight and lengths of *P. clarkii* sampled from different locations.

Sampling Sites	Sample Number	Body Weight (g)	Body Length (cm)
Guangdong (GD)	100	27.61 ± 3.76	7.12 ± 0.69
Hunan (HN)	100	26.48 ± 3.20	7.11 ± 0.47
Hubei (HB)	100	30.74 ± 3.81	8.61 ± 0.56

GD = Guangdong; HN = Hunan; HB = Hubei.

**Table 3:** Mineral element contents (mg·kg^−1^, of dry weight) of *P. clarkii* sampled from different locations.

Element	GD (*n* = 20)	HN (*n* = 20)	HB (*n* = 20)
Ba	1.48 ± 0.35 ^b^	2.49 ± 0.27 ^a^	0.86 ± 0.17 ^c^
Ca	1189.30 ± 119.21 ^a^	936.0 ± 204.84 ^b^	1199.00 ± 70.11 ^a^
Cd	0.0123 ± 0.003 ^b^	0.026 ± 0.008 ^a^	0.014 ± 0.008 ^b^
K	18,347.50 ± 1019.19 ^b^	20,424.00 ± 4569.34 ^a^	19,318.00 ± 438.13 ^ab^
Al	44.30 ± 9.06 ^a^	27.17 ± 5.48 ^b^	15.40 ± 7.85 ^c^
Mg	1533.50 ± 63.77 ^b^	1570.90 ± 44.22 ^a^	1573.00 ± 48.13 ^a^
Mn	14.84 ± 3.06 ^b^	19.87 ± 1.66 ^a^	5.33 ± 2.14 ^c^
Na	2720.50 ± 205.57 ^c^	3482.00 ± 195.95 ^a^	3045.50 ± 144.21 ^b^
Sr	1.102 ± 0.295 ^c^	2.204 ± 0.304 ^a^	1.628 ± 0.169 ^b^
Fe	68.18 ± 10.32 ^a^	56.83 ± 3.91 ^b^	30.42 ± 9.77 ^c^
Cu	21.35 ± 2.84 ^b^	30.40 ± 2.67 ^a^	17.66 ± 1.08 ^c^
Se	0.540 ±0.017 ^b^	0.585 ± 0.025 ^a^	0.515 ± 0.021 ^c^
Zn	77.03 ± 3.67 ^a^	72.99 ± 2.92 ^b^	76.60 ± 1.62 ^a^
As	3.50 ± 0.80 ^a^	1.64 ± 0.45 ^b^	2.13 ± 1.16 ^b^

Values in the same row with different superscript letters are significantly different (*p* < 0.05). GD = Guangdong; HN = Hunan; HB = Hubei.

**Table 4:** Linear discriminant analysis of stable isotope and/or trace element profiles of *P. clarkii* from different geographic origins.

Geographical Origin	Predicted Group (Original/Cross−Validated)			Correctly Classified% (Original/Cross–Validated)
	GD	HN	HB	
**A. Stable isotopes**				
**GD (*n* = 20)**	17/17	3/3	0/0	85/85
**HN (*n* = 20)**	1/1	17/17	2/2	85/85
**HB (*n* = 20)**	0/0	4/4	16/16	80/80
**Total**				83.3/83.3
**B. Trace element profiles**				
**GD (*n* = 20)**	18/18	1/1	1/1	90/90
**HN (*n* = 20)**	1/1	19/19	0/0	95/95
**HB (*n* = 20)**	1/1	0/0	19/19	95/95
**Total**				93.3/93.3
**C. Stable isotopes and elements combined**				
**GD (*n* = 20)**	19/18	1/1	0/1	95/90
**HN (*n* = 20)**	1/1	19/19	0/0	95/95
**HB (*n* = 20)**	0/1	0/0	20/19	100/95
**Total**				96.7/93.3

GD = Guangdong; HN = Hunan; HB = Hubei.

## Data Availability

The datasets generated during or analyzed during the current study are available from the corresponding author on reasonable request.
